# Impact of congestive heart failure on severe sepsis and septic shock survivors: outcomes and performance status after 1-year hospital discharge

**DOI:** 10.1186/cc11007

**Published:** 2012-03-20

**Authors:** M Alkhalaf, N Abd-Aziz, Y Arabi, B Tangiisuran

**Affiliations:** 1 School of Pharmaceutical Sciences, Penang, Malaysia; 2 University Technology MARA, Puncak Alam, Malaysia; 3 National Guard Hospithal, Riyadh, Saudi Arabia

## Introduction

The objective of this study was to evaluate the impact of CHF on severe sepsis and septic shock survivor outcomes after 1 year of hospital discharge.

## Methods

A retrospective cohort and cross-sectional study was conducted at a tertiary-care hospital in Saudi Arabia. All patients (≥18 years) with severe sepsis/septic shock admitted for more than 1 day to the medical-surgical and trauma ICU between April 2007 and March 2010 and alive at hospital discharge were included in the study. Patients who died during admission, could not be contacted and with multiple ICU admission within the same hospitalization were excluded. Data were collected using the electronic ICU database, hospital information system and systematic review of medical records to determine hospital outcomes and performance status pre sepsis. Assessment of the vital status and performance at 1-year hospital discharge were performed via structured telephone interviews using the Karnofsky Performance Status Scale.

## Results

A total of 195 hospital survivors from 364 patients were included in the final analysis. More than 70% of severe sepsis/septic shock with congestive heart failure cases died, 70% of them dead within 3 months. Patients with CHF as compared to patients without CHF had a higher percentage of comorbidity disease (*P *< 0.01) and poor performance status (*P *< 0.05). The majority of these patients (85.7%) who were older (*P *< 0.001), and required a higher dose of dobutamine (*P *< 0.0001), had higher urine output (*P *< 0.001) and prolonged INR (*P *< 0.01) were unable to care for self at 1 year of hospital discharge. Survivors with CHF who died (OR 4.7, CI 1.52 to 14.33) had higher dose of dopamine (*P *< 0.045) and poor performance status pre sepsis (*P *< 0.028).

## Conclusion

About three-quarters of survivors of severe sepsis/septic shock with congestive heart failure died after 1 year of hospital discharge. Many of them (70%) died within 3 months of hospital discharge. The majority had poor performance status and only 14% were able to carry on normal activity at 1 year after hospital discharge. These data highlight the need for different strategies to care for sepsis survivors with congestive heart failure.

